# Beyond Red Pen: Comparing the machine vs human grading of reflective assignments on clinical reasoning in Dermatology undergraduate students

**DOI:** 10.12669/pjms.42.5.12376

**Published:** 2026-05

**Authors:** Lubna Rani Faysal, Shabana Ali, Mahwish Aftab Khan, Humaira Fayyaz Khan

**Affiliations:** 1 Lubna Rani Faysal, Department of Dermatology, Faculty of Medicine and Health Sciences, Riphah International University, Islamabad, Pakistan; 2 Shabana Ali, Department of Anatomy, Faculty of Medicine and Health Sciences, Riphah International University, Islamabad, Pakistan; 3 Mahwish Aftab Khan, Department of Dermatology, Faculty of Medicine and Health Sciences, Shaheed Zulfiqar Ali Medical University, Islamabad, Pakistan; 4 Humaira Fayyaz Khan, Department of Physiology, Faculty of Medicine and Health Sciences, Riphah International University, Islamabad, Pakistan

**Keywords:** AI in medical Education, Clinical Reasoning, Gibbs’ Reflective Cycle, Reflective Writing, Manual vs Automated Scoring

## Abstract

**Background and Objective:**

The integration of Artificial Intelligence (AI) into medical education has been a significant advancement in recent years. AI-based tools like ChatGPT offer numerous advantages for teaching and student assessment. The objective of this study was to evaluate the effectiveness of ChatGPT in assessing reflective assignments compared to human grading.

**Methodology:**

This quasi-experimental study was conducted at Islamic International Medical College from December 2024 to March 2025. In this study a total of 120 3^rd^ year MBBS students were assigned reflective essays after completing their Dermatology module. The e-assignment focused on reflecting on diagnosing scabies, applying clinical reasoning, and correlating clinical findings with patient history, following the Gibbs Reflective Cycle. Each submission was standardized to 500 words and graded using a structured rubric. First the selected faculty members manually assessed each assignment, and then ChatGPT applied the same rubric for grading of the assignments. The scores were then compared for alignment between human and ChatGPT grading.

**Results:**

It **s**howed a strong correlation between ChatGPT and human scores, with minimal differences of 0.5-1.5 marks in a few cases. Clinical Reasoning scores showed a strong correlation (ρ = 0.678, *p* < 7×10^-9^) with a small effect size (d = -0.283), while Gibbs Cycle scores had an even stronger correlation (ρ = 0.734, *p* < 1×10^-10^) and negligible effect size (d = 0.037). Total scores showed very high correlation (ρ = 0.990, *p* < 1×10^-10^) but a large effect size (d = 3.422), suggesting consistent scoring with systematic differences in absolute values.

**Conclusion:**

ChatGPT serves as a valuable and reliable complement to human assessment, significantly improving grading efficiency, particularly in large-scale evaluations.

## INTRODUCTION

Assessment is key to achieving the learning goals of the teaching-learning process. Although AI-based instructional strategies have been used, AI has now gained attention as an assessment tool. Collaboration between educators and artificial intelligence (AI) is a paramount guide for improving the quality of education.^1^ The educational system must revolutionize teachers and teaching culture in the present AI-driven society. AI has been used in automated customer support services, E-Commerce, Healthcare, and education.

Neural networks are used to train large language models (LLM). ChatGPT, an AI chatbot, uses GPT to perform various Natural Language Processing (NLP) tasks, such as writing and code generation.^2^ Natural language processing enables computers to understand human language phrases and words. Although there are many LLMs, such as Microsoft Copilot, Gemini, and Bard, Open AI-designed ChatGPT models are widely used by students, educators, and the general population. This tool provides a matchless personalized learning experience to the users.^2^,^3^

Assessment is an integral component of medical education as it derives learning. In the present era, use of technology as adjunct to manage the magnanimous task of assessment is not surprising but still challenging.^4^ A problem mentioned with automatic grading is the lack of depth and length of feedback while the focus was more on text production skills, writing and linguistics.^5^ A chatbot powered by artificial intelligence can have human-like conversations through which a user can clarify his queries by asking questions and it can also perform certain functions such as grading assessment items in education and data analysis in research.

After the evolution of AI, natural language processing was applied, but despite many benefits, challenges such as cohesion, coherence and feedback have been documented. However, with the advancement of LLMs, such as ChatGPT, the provision of general tools trained on large datasets to educators was made possible. ChatGPT is trained in data from books, Wikipedia, and internet homepages, and therefore, it is thought to be capable of understanding the given text, making text grading possible even without any training.^6^ Also, the feedback provided by ChatGPT is more elaborate. Due to potential bias in the data being used to train the AI model, there is a possibility of issues with the authenticity of answers. The answers also depend upon the quality and the type of prompts used.^7^

Although teachers’ grading has been considered the cornerstone of the educational systems, there is increased hype about using AI to develop MCQs and grading. This has significant implications for reducing the teacher’s workload and giving free time to focus on other educational tasks such as personalized student support, lesson planning, and curricular reform. Additionally, using ChatGPT can help educators focus on professional development.^8^,^9^

The frequent turnover of faculty members presents challenges in maintaining high grading standards, which may result in student dissatisfaction and diminished interest in their tasks. However, with the emergence of artificial intelligence, this issue can be addressed, contingent upon an adequate understanding of the practical application of large language models for essay evaluation. Considering this background, the present study focuses on the scope of using generative AI models as ChatGPT in assessment, specifically grading written essays. It aims to investigate the extent of agreement on grades and scoring between humans and AI using validated rubric for grading reflective essays on clinical reasoning of undergraduate medical students during their rotation in the Dermatology Department.

## METHODOLOGY

A quasi-experimental study was conducted at Islamic International Medical College from December 2024 to March 2025, following approval from the Institutional Review Committee (Appl. # Riphah /IRC/ 24/1054, December 9, 2024). A total of 120 third-year MBBS students participated after completing their Dermatology module. The universal sampling technique was followed, and every second assignment was chosen out of 120 assignments to remove biases. The Islamic International Medical College follows integrated modular system where students have early clinical exposure and students maintain their e-portfolios. A specific list of tasks has been designed from the first to the final year for writing reflective assignments after identified clinical encounters. The students learn to write reflective essays through workshops, and the faculty members get regular departmental training to grade the reflective essay. For this study the following steps were undertaken.

### Step-1:

All third-year medical students (120 in number) had a batch-wise teaching session on clinical reasoning to diagnose different dermatosis in their Dermatology module. After that, students were given the task of writing a 500 words reflective e-assignment using the framework of Gibbs reflective cycle on clinical reasoning to diagnose Scabies from another dermatosis. The task was to “Reflect on your experience during dermatology clerkship, focusing on a case where you were involved in the diagnosis of scabies based upon the interpretation of clinical findings & correlation with the history.” It was an e-assignment uploaded on MOODLE. After task completion, 57 reflective assignments were selected choosing every alternate assignment to minimize biases related to student achievement levels as high or low achievers. Out of 60 selected assignments, 03 assignments were ignored as these were incomplete.

### Step-2:

For assessment of each reflective assignment, a structured rubric (Annexure-I) was used for the assessment of clinical reasoning (CR) validated by two medical educationists & subject experts (LRF & MAK) & each assignment was scored twice both by the human assessors & by ChatGPT, once for clinical reasoning (CR) according to the rubric & once for the use of Gibbs reflective cycle for writing reflection (Annexure-II). For the human (manual) grading, 03 senior faculty members (an Associate Professor of Dermatology, a professor from basic medical science & an Assistant Professor of Dermatology) LRF (R1), SA (R2), & MAK (R3) respectively graded the reflective assignments. For AI-based assessment (ChatGPT grading), the paid version ChatGPT4 was used (Chat GPT Link: https://chatgpt.com/c/66fce482-ca08-8006-8aa3-6225dc863f85).

**Annexure-I:** Rubric for Assessing Reflection on Clinical Reasoning.

Criteria Clinical Reasoning	Exemplary (Exceeds Expectations) (10)	Proficient (Meets Expectations) (7.5)	Developing (Satisfactory) (5)	Beginning (Needs Improvement) (2.5)
Interpretation of Clinical Findings & correlation with history	• Accurately identifies all classic signs of scabies (e.g., burrows, nodules, papules & plaques excoriations with characteristic distribution pattern), correlating them with patient history and risk factors like close living conditions.	• Identifies most signs (e.g., burrows and excoriations), but misses or misinterprets fewer common signs (e.g., nodules).	• Recognizes some key signs (e.g., excoriations) but misses or misinterprets important indicators (e.g., burrows, nodules & distribution pattern)	• Fails to identify key signs of scabies, leading to misdiagnosis or confusion with another condition (e.g., eczema)
	• Correlates with history of Itching, nocturnal aggravation & h/o itching in close contacts	• Correlates history of itching with other signs & takes family history.	• Addresses history of itching, partial correlation with other signs & no mention of close contacts.	• Fails to correlate the history itching with signs of the disease.

***Task:*** Reflect on your experience during dermatology clerkship, specifically focusing on a case where you were involved in the diagnosis of scabies based upon the interpretation of clinical findings & correlation with history. Use Gibbs cycle as a framework for reflection writing. Use evidence from the real patients where required.

**Annexure-II:**
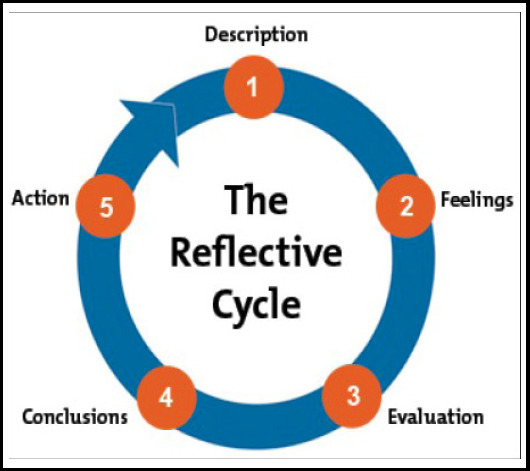
Gibbs Reflective Cycle Framework for reflection writing.

The pre-training of the ChatGPT involved the processing with the prompts & deletion of the redundant characters. With iterative rounds, various steps were taken, such as fine-tuning the prompts and adjustments in the rubric criteria, to improve ChatGPT’s performance over time. The pre-training stage helped to prepare the model to refine both input and output components to learn linguistic patterns, desirable content, sequence of the reflective cycle & rubric for clinical reasoning. The input-output format was set to create similarity for ChatGPT to produce compatible results. The model was trained with five assignments followed by a review process of the results. The results were discussed for the quality and response achieved by using various prompts. Finally, two prompts were selected: Score the assignment out of 10 according to the rubric for clinical reasoning on Scabies’ and ‘Score the assignment out of 10 for use of Gibbs cycle of reflection.’ The responses were noted, followed by grading.

### Step-3:

The data was collected on a Microsoft Excel Sheet for analysis with the help of HK (R4) & AR (R5). At first, the difference between human and machine grading was chalked out for clinical reasoning and then for the use of Gibbs for reflection primarily done by the LRF (R1) & SA (R2). Later, the data was shifted to SPSS for analysis. Nonparametric tests followed the normality assessment. Correlational analysis was done to find the correlational coefficient.

## RESULTS

The results demonstrate a comparative analysis of human versus machine grading of reflective assignments of third-year MBBS students. The difference between the human and ChatGPT grading was assessed for clinical reasoning (CR), the use of Gibb’s cycle for writing reflection, and total scores. Out of 57, ChatGPT gave higher scores in 44 essays compared to manual grading, where 18 were graded with higher scores through manual grading while five essays had no difference. The results generated were categorized into four groups: no difference, negligible, moderate, and major difference (Fig.2). The maximum agreement, “no difference” in grading (12), was found in reflective writing using Gibb’s cycle, while it was the least (5) for the total score of the students. It was also seen that a “negligible difference” in grading was present in reflective writing and CR grading, while a “moderate difference” was more commonly found in total scores and CR. Only total scores showed “maximum difference” in grades (14), Fig.2.

The p-values are < 0.05, indicating non-normal distributions for CR, reflective writing using the Gibbs cycle and Total scores. The mean and standard deviation of the scores is close, with ChatGPT CR scores slightly higher on average. The effect size (Cohen’s d value) was least (-0.037), small to medium (-0.283) and large (-3.42) for reflective writing skills, CR and total scores, respectively, with a trend of difference between human and machine grading (Table-I). In contrast, the negative values are due to slightly higher ChatGPT scores.

**Table-I:** The normality test and descriptive statistics, Spearman rank Correlational coefficient and Mann-Whitney test results for all three parameters used for grading reflective essays.

Test	Shapiro-Wilk Test		Mean ± Std		Effect size Cohnd	Spearman correlation	P-Value	Mann-Whitney U
Parameter	Manual grading P-value	ChatGPT grading P-value	Manual grading	Chat GPT grading				
Clinical reasoning	0.00037	0.00002	7.46 ±0.94	7.72 ±0.91	-0.283	0.68	7e-9	0.09
Using Gibbs cycle	0.00051	1.658e-7	7.38 ±0.98	7.42 ± 1.10	-0.037	0.73	1e-10	0.54
Total score	0.00032	0.00021	15.19±1.77	22.92± 2.66	-3.42	0.99	1.38	0.0000

**Fig.1:**
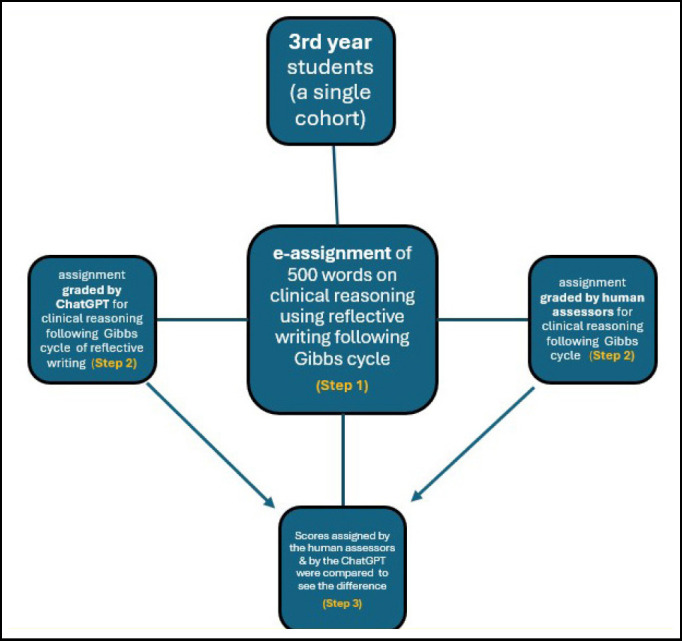
Representation of steps undertaken for the study.

Due to non-normal data distribution, non-parametric tests (Table-I) were used for the correlation analysis. For CR and reflective writing, there was a nonsignificant difference between grading patterns (Fig.1), but a significant difference was found in total score patterns (p<0.001), revealing that total ChatGPT scores are systematically higher than manual scores. The analysis shows that while CR, reflective writing and total scores are highly correlated between manual and ChatGPT scoring, the first two scores show more consistency in absolute values, and the total scores show a systematic difference in scale while maintaining very strong relative agreement.

**Fig.2:**
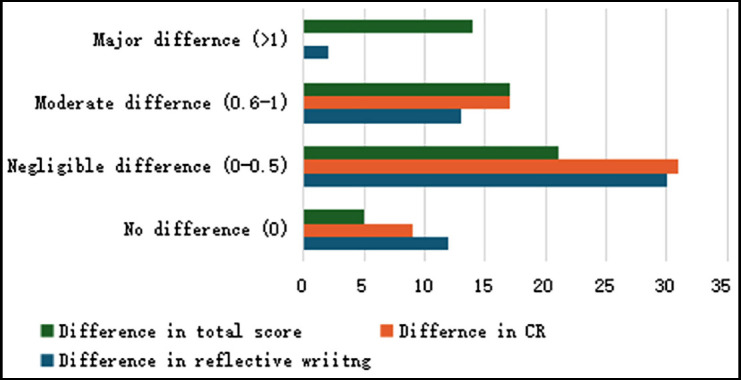
Showing the difference in scoring between human and ChatGPT grading.

**Fig.3:**
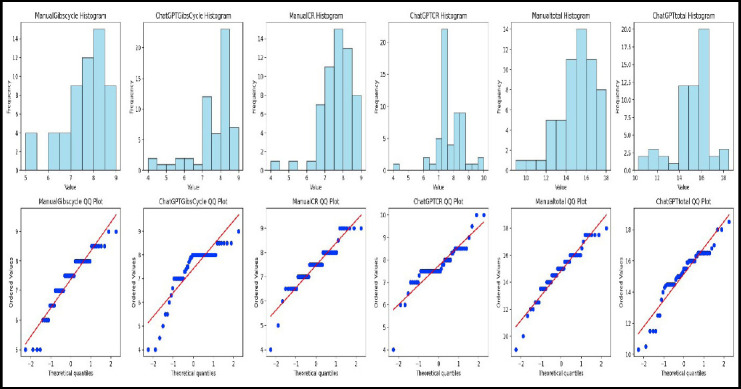
The histogram and QQ plots show the data distribution for all parameters.

A strong positive correlation of 0.678 and 0.734 and a very strong correlation of 0.990 with a highly significant p-value (<0.001) was found for CR, reflective writing and total score, respectively. This indicates a strong monotonic relationship between Manual and Chat GPT Score (Fig.3). The strong Spearman correlation confirms that ChatGPT scores align well with manual scores in terms of ranking. These findings indicate that ChatGPT has considerable promise as a dependable scoring tool, especially in ensuring consistent relative assessments across various domains. The very low p-values from all correlation analyses offer strong statistical confidence in these relationships, significantly reducing the likelihood that these correlations arose by chance.

## DISCUSSION

The findings of our study strongly highlight the potential of ChatGPT as a reliable and consistent scoring tool. The analysis demonstrated a strong positive correlation between ChatGPT-generated scores and human assessors’ scores across all domains, particularly in the assessment of Clinical Reasoning (CR) and Gibbs’ Reflective Cycle. The correlation coefficients (ρ = 0.678 for CR, ρ = 0.734 for Gibbs Cycle, and ρ = 0.990 for total scores) indicate a high level of agreement between AI and human scoring, with particularly strong alignment seen in the overall scores. Interestingly, the negligible effect size observed for Gibbs’ Reflective Cycle assessment (Cohen’s d = 0.037) reflects near-perfect consistency between manual and ChatGPT scoring. This suggests that the ChatGPT is particularly well-suited for assessing reflective writing that adheres to a structured framework, such as Gibbs’ Reflective Cycle, where specific stages are clearly delineated.

Despite the promising correlations, the study revealed some areas that merit further exploration. The effect size for CR was small (Cohen’s d = -0.283), suggesting a slight tendency for ChatGPT to assign higher scores compared to human assessors, though the difference was modest. This difference could be attributed to the ChatGPT inclination to reward structured writing that closely follows the rubric, suggesting that the human scorers may infer context and latent clinical reasoning cues that AI models sometimes underweight. Further refinement of the assessment criteria could potentially mitigate this issue.

In a recent study, Quah et al., evaluated ChatGPT (GPT-4) as an automated essay scorer for written examinations in Oral & Maxillofacial Surgery for undergraduate dental students. The ChatGPT demonstrated strong alignment with human scorers when detailed rubrics were applied. The key findings of the study were strong correlation with human grading for one essay question (r = 0.829, *p* < 0.001), moderate correlation for the second (r = 0.599, *p* < 0.001) & high inter-rater reliability (ICC 0.794–0.858). The AI showed high consistency, with ICC values ranging from 0.794–0.858 and Cronbach’s α between 0.881–0.932. For one essay question, ChatGPT’s scores were statistically indistinguishable from manual grading, although it applied stricter scoring thresholds in another question. These findings indicate that ChatGPT can conduct in-depth, rubric-driven evaluation comparable to trained faculty assessors, particularly when assessment criteria are explicitly defined.^10^ The study presents the strong direct evidence of ChatGPT as an evaluator/assessor in medical education.

Another study compared ChatGPT, human evaluators, and Grammarly in assessing 232 writing samples from Asian English learners. ChatGPT’s accuracy evaluation showed a strong correlation with human evaluation (ρ = 0.79), indicating comparable performance. It also demonstrated a slightly higher correlation with writing scores (ρ = -0.63) than human evaluators (ρ = -0.58), suggesting marginally better prediction of writing proficiency. Compared to Grammarly, ChatGPT exhibited stronger correlations with human evaluation & aligned more closely with expert human judgments.^11^ In a study in orthodontics ChatGPT was found superior to Google Brad in assessing the accuracy of AI Models in Orthodontic knowledge. Across all orthodontic domains tested, ChatGPT-4 scored higher than google Brad.^12^

A study conducted at the University of Hong Kong explored the use of a Chatbot for evaluating 254 technical progress reports from engineering students. Correlation tests compared Chatbot vs teacher scores across holistic and analytical categories. The results showed a moderate correlation in overall scores (r = 0.424) and varied correlations across analytical domains: stronger for language (r = 0.364) and organization (r = 0.316), but weaker for task fulfillment (r = 0.275) and formatting (r = 0.186). The findings suggest that while Chatbots hold potential for automated essay scoring, challenges remain in accurately assessing specific criteria.^13^

A recent study on GPT-4’s performance in grading 60 master’s-level political science essays highlights both its potential and limitations. While GPT-4 showed consistency in average scores, it often failed to align with human judgment. Prompt engineering did not improve accuracy, as the model focused more on generic qualities like language rather than specific grading criteria. Notably, interrater reliability with human assessors was low, emphasizing the need for further development to improve AI’s adaptability and alignment with academic assessment standards.^14^ In contrast to the findings of our study, the prompt engineering and training of the ChatGPT proved to be highly effective in our research. With the refinement of the structured grading process and adherence to a rubric, the alignment between human assessors and ChatGPT showed significant improvement.

A study conducted by Ngoc My Bui & Jessie S. Barrot examined the efficacy of ChatGPT as an AES (Automated Essay Scoring) system by comparing it to an experienced human rater. Overall findings indicate that ChatGPT’s scoring did not closely match that of an experienced human rater and lacked the ability to maintain consistent scoring due to various factors, like ChatGPT’s scoring algorithm, training data, model updates, and inherent randomness.^15^ However, the authors have recommended recalibration with the training data and scoring algorithm, allowing ChatGPT to distinguish quality from poorly written texts.

Another study used ChatGPT (GPT-3) to grade open-ended questions answered by 42 industry professionals in technical training. The effectiveness of ChatGPT was evaluated by comparing its corrections and feedback against subject matter experts. The results suggested that ChatGPT has the capability to identify the fine differences like subject matter experts.^16^

Another research paper has highlighted a ChatGPT-based assessment system to grade exams as an effective tool, particularly in software engineering and computer science courses. ChatGPT has proven effective in accurate grading, identifying knowledge gaps, and providing instant feedback, thereby enhancing learning and reducing instructor workload. It offers consistent, cost-effective, and unbiased assessments with detailed justifications. However, its effectiveness depends heavily on prompt design, highlighting the need for careful prompt engineering.^17^

The study by Li et al. examined the practical utility of ChatGPT as an AI-assisted marking tool for written assessments across different task types, including coding-based and reflective assignments. They concluded it as a reliable adjunct to the human assessors considering consistent & objective evaluation on pre-defined criteria made by ChatGPT. proving its potential in assessment.^18^

A study on ChatGPT in English Language Teaching (ELT) assessment highlighted its benefits, including simplified processes, customization, real-time automation, and consistent scoring across locations. However, it also raised ethical concerns and stressed the need for human validation to ensure fairness and accuracy.^19^ The studies have proposed a comprehensive LLM-based grading system to enhance the entire assessment process, not just scoring, but also the rubric design, feedback generation, and post-grading review. The LLM-based grading has strong potential when it is designed to mirror the complex, iterative, judgment-based workflow of human teachers.^20^,^21^ All these studies support the use of ChatGPT in grading, emphasizing the strong alignment between human assessment and ChatGPT based assessment. The issues of in-depth insight like human assessors can be addressed with prompt training & use of structured rubrics for robust & uniform assessment. ChatGPT excels not only as an efficient assessment tool but also as a reliable source of constructive feedback and for item analysis and detection of item flaws. Its ability to provide consistent, objective evaluations helps eliminate the biases and inconsistencies often associated with human subjectivity.^22^,^23^

### Strength of the study:

The strength of our study is to be the first study at national level to explore the effectiveness of ChatGPT grading in comparison with human grading in medical education to assess the clinical reasoning with promising results.

### Limitations:

It is a single institutional study based on assessment of one dermatosis, to enhance the validity of the results, it is advisable to consider conducting additional analyses with variety of topics at multiple institutes. It can be further improvised by assessing inter-rater reliability, employing a larger sample size to improve statistical power, or executing cross-validation with alternative datasets to ensure generalizability.

## CONCLUSION

The findings of our research paper strongly suggest that ChatGPT holds substantial potential as an adjunct tool for scoring reflective assignments, particularly when large-scale assessments are required. Its application can significantly alleviate the burden on faculty members and enhance the objectivity of scoring. However, it is crucial to recognize that AI-generated assessments should complement rather than replace human evaluation, especially when deeper analysis is required.

### Future recommendation:

Studies are recommended by incorporating a more diverse sample of reflective assignments, covering various clinical scenarios and disciplines. It would provide a more comprehensive understanding of ChatGPT’s applicability as an assessor across different areas of medical education for optimizing its integration in educational assessment frameworks.

### Authors’ Contribution:

**LRF:** Conceived the idea, designed the study and did manuscript writing & editing, is responsible for integrity of research.

**LRF, SA, MAK:** Did data collection & compilation. Critical Review.

**SA, HF:** Literature search, statistical analysis & editing.

All authors have read the final version and approved it. Theya re also accountable for the integrity of the study.
